# Unraveling Glypican-3: From Structural to Pathophysiological Roles and Mechanisms—An Integrative Perspective

**DOI:** 10.3390/cells14100726

**Published:** 2025-05-15

**Authors:** Qianling Piao, Xiaona Bian, Qi Zhao, Luguo Sun

**Affiliations:** National Engineering Laboratory for Druggable Gene and Protein Screening, Northeast Normal University, Changchun 130117, China; piaoql878@nenu.edu.cn (Q.P.); bianxn@nenu.edu.cn (X.B.); zhaoqi@nenu.edu.cn (Q.Z.)

**Keywords:** Glypican3 (GPC3), structural features, biological functions, cancer

## Abstract

*Glypican3* (*GPC3*), initially cloned from rats 40 years ago, deeply participates in the development and homeostasis of multiple tissues and organs. Dysregulation of GPC3 is associated with cancerous and noncancerous diseases. Loss of the function of GPC3 leads to Simpson–Golabi–Behmel syndrome (SGBS), which is characterized by pre- and postnatal overgrowth. However, GPC3 exerts both promotive and inhibitory roles in cancer development. Recent studies suggest that the dual roles of GPC3 in cancer may be attributed to its structural features. This review comprehensively summarizes the structural features, pathophysiological functions, and underlying mechanism of GPC3 and finally discuss the relationship between its structural modification and functions, aiming to provide a theoretical basis for the development of novel therapeutic strategies targeting GPC3 to treat diseases including cancer.

## 1. Introduction

Glypican (GPC) family belongs to the heparan sulfate proteoglycans (HSPGs) and comprises six mammalian members (GPC1 to GPC6) identified to date [1,2,3,4,5,6], with two additional members, Dally and Dally-like protein (DLP), found in Drosophila [7,8]. These members of the GPC family share conserved structural features in their peptide strands: an approximately 60–70 kDa protein with a secretory signal peptide at the N-terminus and a C-terminal hydrophobic region that signals for the addition of glycosylphosphatidylinositol (GPI), anchoring the GPC proteins to the cell surface [9]. Additionally, the C-terminus undergoes heparan sulfate (HS) chain modification, typically occurring within the final 50 amino acids of the C-terminus adjacent to the cell membrane [2]. The modification of HS chains endows GPCs with the ability to recruit multiple ligands, including growth factors, to the cell membrane. While the GPC family members show moderate amino acid sequence homology [2], the positions of 14 cysteine residues are conserved, suggesting a similar three-dimensional structure across glypicans [10]. To date, the crystal structures of human GPC1 (PDB entry 4ACR and 4YWT) [2,3], GPC2 (PDB entry 6WJL and 7T62) [4,5], GPC3 (PDB entry 7ZAW) [6], and DLP (PDB entry 3ODN and 6XTZ) [7,8] have been resolved, and the N-terminal core domains all revealed a similar elongated cylindrical form and a completely α-helical architecture, while the C-terminal 80 amino acid linker region is highly flexible [3]. The flexibility of the C-terminal region presumably allows great freedom for glypicans to reorient to accommodate binding to receptors and other signaling molecules, generally with the participation of the HS chains.

*GPC3* mRNA was first cloned in rat intestinal epithelial cells in 1988, designated OCI-5 [1]. The human *GPC3* gene was first identified in 1996 in patients with Simpson–Golabi–Behmel syndrome (SGBS), and its mutations are strongly implicated in the syndrome’s pathogenesis [2]. Afterwards, the human *GPC3* gene was mapped to the human X chromosome (Xp26) [3], and the first human *GPC3* mRNA was cloned from gastric cancer cells in 1997 [4]. GPC3 is highly expressed in embryonic tissues, suggesting a role in embryonic development, and is typically expressed at low levels in adult tissues such as the heart, lungs, kidneys, and ovaries, with even lower levels in skeletal muscle, pancreas, small intestine, and colon [5]. In contrast, GPC3 is markedly up-regulated in various solid tumors, including hepatocellular carcinoma (HCC), non-small cell lung cancer, testicular and ovarian yolk sac tumors, melanoma, ovarian clear cell carcinoma (OCCA), specific gastric cancer (GC) types, esophageal squamous cell carcinoma (ESCC), testicular germ cell tumors, colorectal cancer, and renal rhabdoid tumors. GPC3 has been affirmed to be pro-oncogenic in a variety of malignancies, particularly due to its oncogenic role, and the underlying mechanisms in HCC have been extensively documented [6]. Thus, GPC3 has emerged as a promising novel biomarker and therapeutic target for HCC, with numerous targeting strategies being developed in recent years [7,8,9,10].

Conversely, GPC3 expression is down-regulated in certain types of cancers, such as ovarian cancer, cholangio-carcinoma, gliomatosis cerebri, and mesothelioma, where it appears to function as a tumor suppressor. So far, the investigations into the anti-oncogenic mechanisms of GPC3 are still limited; thus, the application of GPC3 in cancer treatment still faces significant challenges.

Taken together, GPC3 plays important roles in individual development and in both cancerous and noncancerous diseases. While the dual role of GPC3 in cancer development is recognized, the mechanism governing this duality remains elusive. Emerging studies suggest that the structural features of GPC3 are closely related to its functions. This review aims to summarize the structural features as well as the pathophysiologic roles of GPC3 and their underlying mechanisms. Ultimately, it seeks to provide a fundamental overview of how GPC3’s structural features dictate its functionality, based on the limited existing literature. We hope this review will provide a theoretical basis for new therapeutic strategies targeting GPC3 to treat diseases including cancer.

## 2. The Structural Features of GPC3

**Figure 1:**
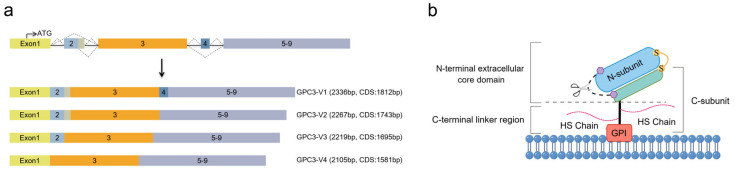
Schematic diagram of *GPC3* splice isoforms and GPC3 structure. (**a**) The comparison of the four alternative splicing isoforms of *GPC3* mRNA, V1, V2, V3, and V4. (**b**) The structure of GPC3–V2 protein, with a 70 kDa core protein that is anchored to the exocytoplasmic surface of the cell membrane via a GPI anchor added to the C-terminus and carries two HS side chains attached at the highly conserved Ser495 and Ser509 positions. The initial translated peptide undergoes hydrolytic cleavage between Arg358 and Ser359 by Furin-like convertase, producing an approximately a 40 kDa N-subunit and a 30 kDa C-subunit, which are linked to each other by one or more disulfide bonds. V, variant; CDS, coding sequence; GPI, glycosylphosphatidylinositol; HS, heparan sulfate.

The NCBI database indicates that the *GPC3* gene has four mRNA variations through alternative splicing (Figure 1a). The first cloned mRNAs from rat and human sources both correspond to variant 2 (V2), which has been the main isoform in subsequent studies, indicating that V2 may be the predominant and functional variant of *GPC3* in cells. The differences among these *GPC3* variants mainly reside in the second and fourth exons (Figure 1a), with V1 being the longest, encompassing all exons, while V2 lacking a 69-base pair long exon 4.

Current structural insights into the GPC3 protein primarily pertain to V2. *GPC3* V2 encodes a 580-amino-acid (aa)-long initial translation product, in which the C-terminal hydrophobic domain is excised and added with GPI for being anchored to the outer cell membrane, a process common to other GPC family members. The C-terminus of GPC3 has two HS side chains attached to the highly conserved Ser495 and Ser509 residues [11]. In addition, the GPC3 protein is hydrolytically cleaved between Arg358 and Ser359 by Furin-like convertase [12] (Figure 1b), producing an N-terminal subunit of about 40 kDa and a C-terminal subunit of approximately 30 kDa, which contains the HS chains. These two subunits are interconnected by one or more disulfide bonds [6]. Upon the reduction of disulfide bonds, the mature GPC3 protein appears as two separate subunits with different molecular weights. The crystal structure showed that GPC3 has a more curved shape compared to fly DLP and human GPC1. A schematic diagram of the mature GPC3 structure is depicted in Figure 1b.

De Cat B et al. propose that GPC3 is synthesized as a pro-protein in the endoplasmic reticulum (ER), where it is transferred to GPI; then, GPI-anchored GPC3 shuttles to the Golgi complex for HS modification and convertase cleavage into two subunits. The mature GPC3 eventually travels to the cell surface membrane [13]. Asn124 and Asn241 in human GPC3 are predicted to be the N-glycosylation sites. As one of the corresponding N-glycosylation sites, Asn79 in GPC1 is shown to assist both in targeting and associating the Gpc1 protein to the consensus lipid raft domains of the cell surface and in GAG substitution on the C-terminal domain [14]. Asn124 and/or Asn241 are supposed to play similar roles in GPC3. In addition to the GPI-mediated membrane-bound form of GPC3, there is also soluble GPC3 [13,15,16], which is detectable in the medium of cells cultured in vitro and in the serum of patients with HCC [16]. Filmus J’s group has demonstrated that the lipase Notum can cleave the GPI anchor and shed GPC3 from the membrane, and Notum is especially active in the extracellular environment [15]. Recently, we identified the individual soluble N-subunit of GPC3 in the culture medium of cell lines, indicating that the N-subunit of GPC3 may elicit functions by itself.

**Figure 2:**
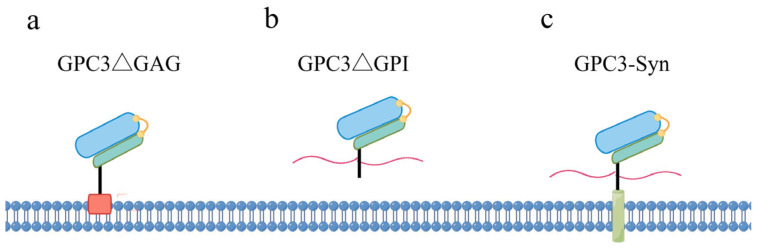
Schematic diagram of the four mutant forms of GPC3. (**a**) GPC3 mutant lacking HS chains; (**b**) GPC3 mutant that becomes soluble by deletion of the GPI anchor site; (**c**) membrane-anchored GPC3–Syndecan fusion protein.

The structural modification of GPC3 introduced above can be modified by artificial targeted mutagenesis, as illustrated in Figure 2. Mutations at Ser495 and Ser509, resulting in the GPC3ΔGAG mutant (Figure 2a), prevent HS chain modification. The Arg355 and Arg358 mutations, referred to as RR-AA, disrupt the convertase cleavage site, leading to a single, uncleaved polypeptide chain rather than two disulfide-bonded subunits. Removing the last 30 residues, including the GPI anchoring sequence (GPC3ΔGPI), hinders GPI attachment and hence generates secreted soluble GPC3 (Figure 2b). In contrast, the fusion of GPC3 with the transmembrane domain of the Syndecan protein (GPC3–syn) anchors GPC3 to the cell membrane, preventing its shedding by lipases to generate soluble GPC3 (Figure 2c). Given the profound impact of these structural features on the pathophysiological functions of GPC3, the utilization of these mutants is an effective strategy to delineate the structure–function relationship of GPC3.

## 3. Physiological Functions and Mechanisms of GPC3

GPC3 is highly expressed during embryogenesis, and its loss-of-function mutations are closely related to SGBS, a congenital genetic disorder, suggesting essential roles of GPC3 in human development. Subsequently, researchers have utilized *GPC3* knockout (*GPC3*-/-) mice to further elucidate its physiological functions and potential mechanisms in both developmental and adult tissues.

### 3.1. Physiological Functions of GPC3

GPC3 is predominantly expressed during embryonic development but is down-regulated after birth [1], indicating its involvement in morphogenesis. Consistently, mutations in the human *GPC3* gene cause SGBS, an X-linked disorder characterized by pre- and postnatal overgrowth [17]. The clinical manifestations of SGBS patients include a distinct facial appearance, macroglossia, cleft palate, syndactyly, polydactyly, supernumerary nipples, cystic and dysplastic kidneys, congenital heart defects, rib and vertebral abnormalities, and umbilical/inguinal hernias [18,19,20,21]. So far, various mutations in the human *GPC3* gene have been identified in SGBS patients, predominantly consisting of point mutations or small deletions encompassing a varying number of exons [22,23,24,25]. The lack of a clear correlation between patient phenotypes and mutation sites suggests that diverse mutations converge on impairing or abolishing GPC3 function, thereby causing SGBS, whereas intra- and inter-familial phenotypic variability may be due to other genetic factors [24]. As loss-of-function mutations of GPC3 result in the overgrowth phenotype of SGBS, GPC3 is supposed to participate in the regulation of cell proliferation and apoptosis during developmental morphogenesis.

The establishment of *GPC3*-/- mouse bolsters the hypothesis that loss-of-function mutations in GPC3 lead to SGBS [26]. *GPC3*-/- mice display most of the clinical features observed in SGBS patients, such as developmental overgrowth, cystic and dysplastic kidneys, abnormal lung development, and so on [26]. Starting from the early stage of kidney development, a persistent increase in the proliferation rate of epithelial cells in the ureteric bud/collecting system has been observed in *GPC3*-/- mice [26]. *GPC3* knockout mice also develop abnormal limb and skeletal development, such as post-axial polydactyly [27] and rib deformities due to delayed endochondral ossification [28]. In addition, Ng A et al. discovered a high prevalence of multiple cardiac malformations in *GPC3*-/- mice, including ventricular septal defects, common atrioventricular canal, double outlet right ventricle, and coronary artery fistulas [29], the latter of which has not been previously reported in SGBS patients. So far, the molecular underpinnings of coronary artery fistulae remain largely unknown, but the authors speculate that its occurrence in *GPC3*-/- mice may be related to excessive coronary artery development during embryogenesis [29].

GPC3 expression is restricted to only a few adult tissues, and its function in normal adult tissues and cells has been less extensively studied. Several studies reveal that GPC3 plays a negative regulatory role in liver regeneration and hepatocyte proliferation; *GPC3* mRNA and protein levels began to significantly increase in the liver following partial hepatectomy, which induced hepatocyte regeneration [30,31]. However, hepatocyte-targeted transgenic GPC3 has been shown to suppress hepatocyte proliferation and liver regeneration after partial hepatectomy [32], as well as greatly attenuate drug-induced hepatomegaly by inhibiting hepatocyte proliferation in mice [33]. Consistently, in vitro studies have shown that blocking GPC3 expression promotes hepatocyte growth [31]. Collectively, these studies illustrate that GPC3 serves as a negative regulator of cell proliferation in normal hepatocytes.

Moreover, GPC3 is positively expressed in human hematopoietic stem/progenitor cells (HSPCs) and *GPC3* knockdown resulted in enhanced proliferation but decreased homing ability of HSPC, as evidenced by a decrease in the primitive HSC pool in bone marrow (BM) and an increase in the proportion of circulating HSPCs in peripheral blood [34]. In human embryonic stem cell (hESC)-derived neural stem cells (hNSC H9 cells), GPC3 expression is also observed, and the down-regulation of GPC3 has been shown to alter hNSC H9 cell lineage potential [35]. In adipocytes, GPC3 interacts with glucose transporter4 (GLUT4) to promote its exocytosis onto the cell surface membrane, and the overexpression of GPC3 promotes glucose uptake [36], suggesting that GPC3 may play a role in the process of glucose metabolism. GPC3 is the most expressed transcript in tracheobronchial epithelial cells, and the decrease in GPC3 transcript levels correlates with the severity of airway obstruction in chronic obstructive pulmonary disease (COPD) patients [37]. The GPC3 protein is detectable in the follicle fluid of women, and it is increased or decreased in endometriosis patients or women with diminished ovarian reserves, respectively, implying roles of GPC3 in the reproductive function of females [38,39].

### 3.2. Mechanisms Underlying GPC3’s Physiological Function

GPC3, a GPI membrane-anchored heparan sulfate proteoglycan, commonly acts as a co-receptor of membrane surface receptors involved in the regulation of ligand signaling, including growth factors. To date, various signaling pathways and molecules have been implicated in GPC3’s regulatory roles in physiological conditions.

Insulin-like growth factor II (IGF-II)-signaling pathway: SGBS shares several clinical features with Beckwith–Wiedemann syndrome (BWS), another overgrowth syndrome [40]. Given that the overexpression of IGF-II is thought to be one of the contributing factors to BWS [41], and since GPC3 has been shown to bind specifically to IGF-II [2,42], it has been proposed that GPC3 may negatively regulate IGF-II activity during embryonic growth by competitively binding to the IGF-II receptor [43]. However, Song HH et al. did not observe an increase in the phosphorylation levels or an activation of downstream IGF-signaling molecules such as insulin receptor substrate-1 (IRS-1) and IGF receptor-1 (IGF1R) in the embryonic tissues of *GPC3*-/- mice. Furthermore, the embryonic overgrowth phenotype of offspring mice from crosses between *GPC3*-/- mice and IRS-1-/- mice was not mitigated, which further demonstrates that embryonic overgrowth due to GPC3 inactivation is not associated with the overactivation of the IGF-II-signaling pathway [44]. Nevertheless, GPC3’s ability to bind IGF-II suggests that it may modulate IGF-II signaling in certain conditions.

Wnt-signaling pathway: While Song HH et al. excluded the involvement of IGF-II in the abnormalities observed in *GPC3*-/- mice, they concomitantly demonstrated that the canonical Wnt-signaling pathway (β-catenin pathway) was overactivated in the embryonic tissues of *GPC3*-/- mice, whereas the non-canonical Wnt signaling pathway (JNK pathway), which typically counteracts the canonical pathway, was significantly suppressed [44]. These findings were then confirmed in vitro, where overexpressed GPC3 in the cells was shown to interact with Wnt5a, potentially facilitating the activation of the non-canonical Wnt-signaling pathway [44]. Thus, embryo overgrowth attributed to GPC3 loss-of-function is more likely associated with the hyperactivation of the canonical Wnt-signaling pathway.

Hedgehog (Hh)-signaling pathway: The protein level of Indian Hedgehog (Hh) is increased and its downstream signaling is enhanced in *GPC3*-deficient mice. Additionally, breeding *GPC3*-deficient mice with Indian Hh-deficient mice results in a significant reduction in the percentage of offspring mice with overgrowth phenotypes, suggesting that the overgrowth abnormalities caused by *GPC3* deficiency or functional inactivation are at least partially due to its inhibition of the Hh pathway [45]. This inhibitory effect is further supported by studies in mouse embryonic fibroblasts (MEFs), where GPC3 is shown to inhibit the Hh-signaling pathway (including both Indian and Sonic Hh) by competing with Patched (Ptc) for Hh binding, which in turn induces Hh endocytosis and degradation [46]. Moreover, during liver regeneration in mice following partial hepatectomy, GPC3 binding to Indian Hh decreases and Hh signaling is enhanced at the peak of hepatocyte proliferation, which also suggests that GPC3 may play an inhibitory role in Hh signaling during hepatocyte proliferation [47]. However, a reduction in Sonic Hedgehog (Shh) mRNA expression and signaling is observed in mutant hearts of *GPC3*-/- embryos [28]. Liu YC et al. found in *GPC3* knockout MEFs that GPC3 is necessary for the Sonic Hh response via controlling a downstream signal transduction step but not by direct binding with Sonic Hh [48], which is contrary to previous observations [42]. Consistently, interfering with GPC3 signaling during airway epithelial cell differentiation induces the down-regulation of the Hh pathway, attested by a decrease in Gli2 leading to reduced ciliogenesis and altered mucin production [37]. Therefore, the regulation effect of GPC3 on Hh signaling is complicated and not yet fully clear.

BMP-signaling pathway: *GPC3* deficiency in embryonic kidney explants abrogates the inhibitory activity of bone morphogenetic protein (BMP) 2 on branch formation, converts BMP7-dependent inhibition to stimulation, and enhances the stimulatory effects of keratinocyte growth factor (KGF) [49]. These alterations may lead to the abnormal morphogenesis of renal branching in *GPC3* knockout mice, indicating that GPC3 is crucial for maintaining BMP-mediated inhibitory signals during embryonic kidney development. Furthermore, abnormal limb and bone development in *GPC3* knockout mice may be also associated with aberrant BMP4 signaling [27].

Unc5D: Uncoordinated-5 receptor D (Unc5D) plays a pivotal role in guiding neurons during radial migration, a process essential for the formation of functionally distinct cortical layers [50]. Crystal structural analysis reveals that Unc5D forms an octameric glycoprotein complex with GPC3, in which four Unc5D molecules pack into an antiparallel bundle, flanked by four GPC3 molecules. Unc5/GPC3 signaling causes cell contact–repulsion in vitro and modulate the migration of cortical neurons and neuroblastoma cells in vivo [50]. The intricate balance of Unc5D–GPC3 interactions is a conserved mechanism in cell guidance, influencing cell migration.

CD81: CD81, a widely expressed tetraspanin transmembrane protein, serves as a signaling platform for various cellular processes [51]. During mouse liver regeneration following partial hepatectomy, both CD81 and GPC3 expressions rise from the second post-hepatectomy day, with their interaction peaking at the end of hepatocyte proliferation [31]. This interaction diminishes the binding between CD81 and hematopoietically expressed homeobox (Hhex), a known transcriptional repressor, thereby releasing Hhex to enter the nucleus and exert transcriptional repression [47]. These findings suggest that GPC3 inhibits hepatocyte proliferation by interacting with CD81, which in turn relieves the inhibitory effect of CD81 on Hhex, preventing hepatocyte over proliferation during liver regeneration.

**Figure 3:**
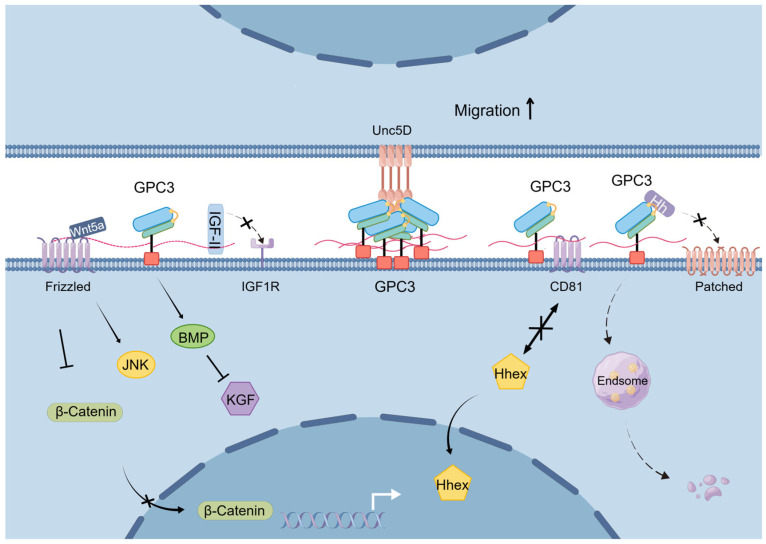
Schematic diagram of the mechanisms underlying GPC3’s physiological functions.

In summary, GPC3 plays an important negative regulatory role in cell proliferation across mammalian embryonic development and in adult tissues, and this regulatory role may be related to its modulation of various signaling pathways (Figure 3).

## 4. GPC3 in Cancer

**Figure 4:**
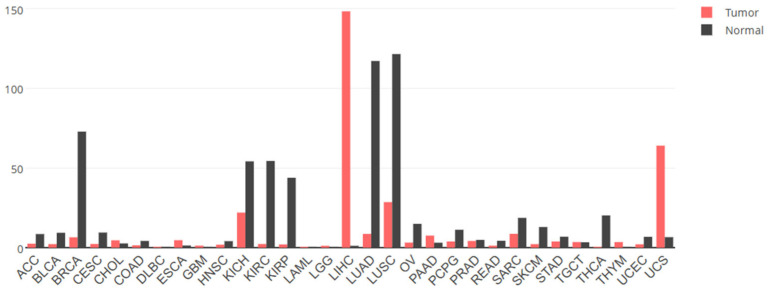
The expression levels of *GPC3* mRNA in different tumor tissues and normal tissues. Data from GEPIA2 (http://gepia2.cancer-pku.cn/ (accessed on 2 April 2025). ACC, adrenocortical cancer; BLCA, bladder cancer; BRCA, breast cancer; CESC, cervical cancer; CHOL, bile duct cancer; COAD, colon cancer; DLBC, large B-cell lymphoma; ESCA, esophageal cancer; GBM, glioblastoma; HNSC, head and neck cancer; KICH, kidney chromophobe; KIRC, kidney clear cell carcinoma; KIRP, kidney papillary cell carcinoma; LAML, acute myeloid leukemia; LGG, lower-grade glioma; LIHC, liver hepatocellular cancer; LUAD, lung adenocarcinoma; LUNG, lung cancer; LUSC, lung squamous cell carcinoma; OV, ovarian cancer; PAAD, pancreatic cancer; PCPG, pheochromocytoma and paraganglioma; PRAD, prostate cancer; READ, rectal cancer; SARC, sarcoma; SKCM, skin cutaneous melanoma; STAD, stomach cancer; TGCT, testicular cancer; THCA, thyroid cancer; THYM, thymoma; UCEC, endometrioid cancer; UCS, uterine carcinosarcoma.

In addition to the sparse research on the physiological functions of GPC3, as mentioned above, the vast majority of scholarly attention has been directed towards its pathological implications in cancer. The expression and roles of GPC3 in cancer are notably tissue-dependent. For instance, data from the cancer RNAseq database indicate that *GPC3* mRNA levels are diminished in breast, kidney, lung and ovarian cancers when compared to their normal counterparts, yet a stark contrast is observed in HCC where *GPC3* levels are markedly elevated relative to normal liver tissues (Figure 4). At the protein level, immunohistochemical analysis reveals that GPC3 expression is reduced exclusively in breast and lung adenocarcinoma tissues when juxtaposed with normal tissue, while it exhibits heightened expression in various cancer types, including HCC and melanomas (as detailed in Table 1). Accordingly, GPC3 has the potential to exert either a pro- or anti-oncogenic effect, contingent upon the specific cancer type.

### 4.1. GPC3 as an Oncogene in Cancers

#### 4.1.1. Oncogenic Roles of GPC3

The up-regulated expression of GPC3 in liver cancer, along with its pro-oncogenic properties, has been explored intensively and extensively. Consequently, GPC3 has emerged as a significant novel biomarker for HCC, complementing the traditional marker alpha-fetoprotein (AFP) [8,73,74]. GPC3 is overexpressed in a higher proportion of HCC patients compared to AFP, which positions it as an optimal target for immunotherapy [8,75]. A range of GPC3-targeted therapeutic strategies are being developed, such as GPC3 vaccines, anti-GPC3 antibodies and immunotoxin, GPC3-targeted aptamers, and various GPC3-directed immunotherapeutic approaches [10,76,77]. Through loss- and gain-of-function studies, it has been established that GPC3 fosters the proliferation, migration, and invasion capabilities of HCC cells while simultaneously inhibits apoptosis [78,79,80,81,82]. Meanwhile, GPC3 also facilitates the epithelial–mesenchymal transition (EMT) in HCC cells, a process closely related to tumorigenesis and metastasis [56,83,84]. Notably, the GPC3 levels in HCC cells and tissues exhibit an inverse correlation with the E-cadherin levels [56,83]. Thus, GPC3 is intricately implicated in the occurrence and development of HCC [85]. Similar oncogenic effects have been reported in other malignancies characterized by elevated GPC3 expression, including ovarian clear cell carcinoma [86], cervical cancer [87], lung squamous carcinoma [88] and gastric cancer cells [89]. In addition, GPC3 expression levels were further increased in drug-resistant tumor cell lines, suggesting its potential role in cancer cell drug resistance. For instance, the down-regulation of GPC3 expression increased the sensitivity of drug-resistant gastric cancer cells to mitoxantrone [90], and the knockdown of *GPC3* increased the sensitivity of ovarian clear cell carcinoma cells to paclitaxel [86]. However, in melanoma, the role of *GPC3* as an oncogene or a tumor suppressor remains controversial despite its up-regulated expression [91].

#### 4.1.2. Pro-Oncogenic Mechanism of GPC3

The majority of the physiological functions of GPC3 involve the inhibition of growth factor signaling, but its pro-oncogenic effects in cancers are accomplished by facilitating the signaling of a variety of growth factors. For example, *GPC3* knockdown leads to the down-regulation of various growth factor mRNA and protein levels in HCC cells [92]. The major growth-factor-signaling pathways through which GPC3 mediates its pro-oncogenic effects are outlined below.

Wnt-signaling pathway: The activation of Wnt signaling is considered to be the predominant mechanism by which GPC3 promotes the growth and development of HCC. GPC3 can stimulate both paracrine and autocrine canonical Wnt signaling in HCC cells via interacting with Wnts and acting as a co-receptor to facilitate Wnt’s binding to the frizzled receptor [82]. Moreover, GPC3 can also activate Wnt signaling by increasing the amount of Wnt on the cell membrane and binding to both Wnt and frizzled receptor to facilitate the formation of signaling complexes [13]. Consistently, immunotoxins targeting GPC3 can impede HCC growth in vivo and in vitro by reducing Wnt expression and repressing Wnt signaling, while those GPC3-targeting immunotoxins that fail to inhibit Wnt signaling cannot effectively inhibit HCC growth [93]. This further substantiates the notion that the augmentation of Wnt signaling is the most critical mechanism by which GPC3 promotes HCC progression.

IGF-signaling pathway: GPC3 has been shown to interact with IGF-II and IGF1R and enhance the phosphorylation and activation of IGF1R in HCC cells, thereby triggering the downstream ERK cascade [85]. GPC3 was also demonstrated by the same research group to reduce IGF-I-mediated IGF-1R ubiquitination and subsequent degradation [94], thereby preserving IGF1R levels and sustaining the activation of ERK pathway. Therefore, the regulatory effect of GPC3 on the IGF-signaling pathway may constitute another essential mechanism underlying GPC3’s role in promoting hepatocellular carcinogenesis [85,94].

Hippo/YAP pathway: *GPC3* knockdown in HCC cells resulted in the down-regulation of both mRNA and protein levels of Yes-associated protein (YAP), and the exogenous addition of YAP-1 can partly rescue the cells from *GPC3* knockdown-induced apoptosis as well as restore the decreased capabilities in proliferation, migration, and invasion [78]. This suggests that the oncogenic effect of GPC3 on HCC is, at least in part, attributed to the up-regulation of YAP and the subsequent activation of the downstream Hippo pathway. Consistent with these findings, a potent GPC3-targeted antibody has been shown to induce G1 phase arrest and thus inhibit the proliferation of HCC cells in vitro and in vivo through interfering with the YAP-signaling pathway [95].

Regulating the tumor microenvironment: GPC3 is up-regulated in the cancer-associated fibroblasts (CAFs) subgroups within the advanced GC and is correlated with poor prognosis in GC patients [96]. In addition, GC patients with CAFs that highly express GPC3 exhibit a higher TIDE (Tumor Immune Dysfunction and Exclusion) score and respond poorly to PD-1 immunotherapy, which could be due to the fact that GPC3, secreted by CAFs, promotes the expression of the immune checkpoint genes, such as PD-L1, TIM3, and CD24, in GC cells [96]. These findings indicate that GPC3, as expressed by the components of the tumor microenvironment, is also involved in promoting cancer development.

Others: In addition to the main GPC3-regulated signaling pathways listed above, there is scattered evidence hinting at GPC3’s potential to modulate other signaling pathways as well. For instance, the inhibition of GPC3 expression in HCC cells leads to the up-regulation of the expression of transforming growth factor-β (TGF-β), which in turn inhibits cell proliferation, blocks cell cycle progression, and induces replicative senescence [80]. Furthermore, sulfate esterase 2 (SULF2) has been demonstrated to enhance FGF2 signaling in HCC cells via elevating GPC3 expression [97]. GPC3 also interacts with hepatocyte growth factor (HGF) and promotes HCC cell migration and motility through the activation of the HGF/c-Met pathway [98].

### 4.2. GPC3 as a Tumor Suppressor in Cancers

#### 4.2.1. Anti-Oncogenic Role of GPC3

GPC3 expression is often silent or decreased in ovarian cancer except clear cell carcinoma types [99], as well as in mesothelioma [66], breast cancer [72], lung adenocarcinoma [71,100], and clear cell renal carcinoma. The ectopic expression of GPC3 in cell lines originating from these cancer types inhibits cancer cell proliferation [66,71,72,99,101] and/or induces apoptosis [11,71]. In addition, GPC3 reduces the migratory and invasive ability of breast [102], ovarian [103], and lung cancers [104] by suppressing EMT. In ovarian cancer, cells that exhibit resistant to platinum-based chemotherapeutic drugs tend to express lower levels of GPC3, suggesting a negative regulatory role for GPC3 in the development of drug resistance [105].

Confusingly, there are also some reports showing GPC3 to act as a tumor suppressor gene in certain tumor types where GPC3 is overexpressed and has been shown to play an oncogenic role. For instance, in HCC cells [106,107], clear cell ovarian carcinoma cells [63], and gastric carcinoma cells [108], GPC3 has been demonstrated to inhibit tumor growth or metastasis. The factors contributing to the conflicting conclusions in these tumor types are not yet clear, but they highlight the complex and context-dependent nature of GPC3’s role in cancer biology. This complexity calls for further research to elucidate the mechanisms underlying the dualistic behavior of GPC3 across different cancer types.

#### 4.2.2. Mechanism of Cancer Inhibition by GPC3

The suppressive effects of GPC3 on certain types of cancers align with its functions in embryonic development and within normal tissues. Indeed, current studies indicate that the molecular mechanisms underlying GPC3’s anti-oncogenic activities partially overlap with the signaling pathways regulated by GPC3 under physiological conditions.

Wnt-signaling pathway: LM3 breast tumor cells, when overexpressing GPC3, can secrete GPC3 to competitively interact with Wnt ligands, thereby preventing their binding to the Wnt receptor and consequently curbing the canonical Wnt-signaling pathway [109]. In addition, GPC3 has been shown to selectively inhibit proliferation and induce apoptosis in certain breast cancer cells and mesothelioma cells by interacting with Wnt5a to trigger non-canonical Wnt/JNK signaling and concomitantly suppress canonical Wnt/β-catenin signaling [13,44]. Therefore, the repression of canonical Wnt signaling and/or activation of non-canonical Wnt signaling represent one of the key mechanisms underlying the anti-oncogenic role of GPC3.

IGF-II-signaling pathway: Sakurai M et al. [63] found that despite its high expression in the ovarian clear cell carcinoma (CCC) subtype of ovarian cancer, *GPC3* knockdown in CCC cells promoted cell proliferation via enhancing IGF-II-mediated activation of the PI3K/Akt pathway. This suggests that the overexpressed GPC3 in CCC may play an inhibitory role by suppressing the IGF-II-signaling pathway.

P38 MAPK pathway: In breast cancer cells, GPC3 has been shown to reverse the EMT, prevent metastatic spread, and induce dormancy at secondary sites by activating the p38 mitogen-activated protein kinase (MAPK) pathway [110].

Hh-signaling pathway: Also in breast cancer cells, Capurro MI et al. have reported that GPC3 is capable of triggering the endocytosis and subsequent degradation of the GPC3–Hh complex, thereby inhibiting Hh signaling [111], just as it does in fibroblast cells [42]. However, it remains to be definitely demonstrated whether GPC3 exerts anti-oncogenic effects in breast cancer by inhibiting Hh signaling.

TGF-β-signaling pathway: In ovarian cancer cells, the down-regulation of GPC3 expression by RNA interference has been shown to elevate the levels of TGF-β2 protein, accompanied by the increase in cell proliferation and tumor growth in vivo. The researchers hypothesize that the increase in TGF-β2 expression contributes to the growth of tumors derived from *GPC3* knockdown in ovarian cancer cells [103].

Others: *GPC3* knockdown in ovarian cancer cells can lead to an increase in the expression of matrix metalloproteinase 2 (MMP-2) and MMP-9, along with a decrease in the level of tissue inhibitors of metalloproteinase 1 (TIMP-1) [103]. The imbalance of MMPs and TIMP-1 might contribute to the enhanced motility and invasion observed in ovarian cancer cells with *GPC3* knockdown [103].

**Figure 5:**
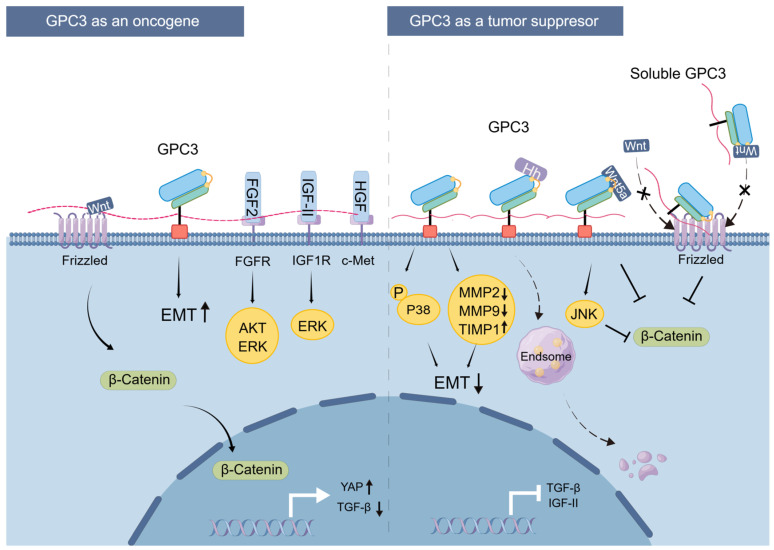
Schematic diagram of GPC3 involvement in cancer development.

Taken together, GPC3 can target the same signaling pathways to exert diametrically opposed effects, thereby promoting or inhibiting cancer progress in different tumor cells. The mechanisms underlying GPC3’s dual role in mediating either pro- or anti-oncogenic effects, respectively, are summarized in Figure 5.

## 5. Relationship Between the Structural Features of GPC3 and Its Functions

What determines that the GPC3 protein can play diametrically opposite roles in distinct contexts? In cancers, the intrinsic factors of the tumor cells, such as genetic background, tissue origin, gene expression profile, etc., may influence the functional manifestation of GPC3. In addition, given the GPC3 protein’s distinct structural features (as depicted in Figure 2), variations in these structural features may also be pivotal in dictating GPC3’s roles. Next, we summarize the studies on the relevance of GPC3’s structural attributes to its functional diversity as follows.

### 5.1. GPC3’s Structural Features and Its Physiological Functions

In terms of the influence of GPC3’s structural characteristics on its physiological functions, Filmus J’s research team has unveiled a wealth of insights through a series of publications. They have highlighted the importance of GPC3’s structure in its inhibition of the Hh pathway, a vital signaling pathway in the development of organisms and tissues. Capurro MI et al., using embryonic fibroblasts in their studies, found that the GPC3ΔGPI mutant, which generates a soluble form but not a membrane-attached GPC3, completely loses the ability to inhibit Hh signaling, underscoring the necessity of membrane attachment for GPC3 to inhibit Hh signaling. In addition, they observed that the GPC3ΔGAG mutant lacking HS chains could still interact with Hh but exhibited a diminished capacity to inhibit the pathway, suggesting that GPC3’s core protein is responsible for Hh interaction, whereas HS chains are indispensable for its inhibitory effect on Hh signaling [46]. The team further revealed that the HS chains of GPC3 are required for GPC3 to interact with low-density lipoprotein receptor-related protein-1 (LRP1), a mediator of the endocytosis and degradation of the GPC3–sonic Hh complex. Therefore, the GPC3ΔGAG mutant, which can interact with sonic Hh but is incapable of binding to LRP1, fails to trigger the GPC3ΔGAG–sonic Hh complex’s endocytosis and degradation, leaving Hh signaling unopposed [111].

Another study from the same research team shows that processing by convertases, which generates two separate subunits linked by disulfide bonds, is essential for GPC3 to inhibit Hh signaling by competing with the Ptc receptor for Hh binding and mediating the endocytosis and degradation of the GPC3–Hh complex. The evidence for this mechanism comes from the observation that the convertase-resistant GPC3 mutant (RR-AA) not only fails to inhibit but actually enhances Hh signaling. This occurs through interactions with both Hh and Ptc via the HS chains, leading to increased Hh binding to Ptc. The researchers propose that the structural alterations in GPC3 due to RR-AA mutation elevates the degree of HS chain sulfation, which provides the larger binding capacity to Ptc as compared with wild-type GPC3 [112]. The aforementioned findings indicate that during embryonic development, the GPC3-mediated inhibition on Hh signaling in certain cells depends on its membrane attachment, its cleavage by convertase to form a two-subunit structure, and the appropriate degree of HS chain sulfation (Table 2).

As for binding with Unc5D, crystal structural analysis demonstrates that the N-linked glycan at Asn241 of human GPC3 is responsible for interacting with the C-mannosylated tryptophans of the Unc5D thrombospondin-like domains, which are indispensable for the formation of octameric GPC3–Unc5D complex [6]. However, the convertase-resistant GPC3 mutant (RR-AA) retains the ability to form an octamer, indicating that convertase cleavage is not required for binding with Unc5D [6]. Together, these structural features enable GPC3 to serve its physiological purpose in the intricate regulatory mechanisms, but they still remain largely unknown.

### 5.2. GPC3’s Structural Features and Its Oncogenic Function

How GPC3’s structural features determine its pro-oncogenic roles has been relatively extensively investigated via utilizing various GPC3 mutants, especially in liver cancer and the activation of Wnt signaling, one of the vital mechanisms underlying its oncogenic role.

When revealing for the first time that GPC3 promotes HCC growth through the activation of the canonical Wnt pathway, Filmus J’s team also elucidated that GPC3 lacking HS chains, the GPC3ΔGAG mutant, retains the ability to interact with Wnt3a and activate its signaling to promote HCC growth, suggesting that the HS modification at the C-terminus of GPC3 is not required for its activation of Wnt signaling and HCC-promoting effects [82]. Furthermore, based on the crystal structures of the hGPC1 and DLP proteins bound to the lipid of Wnt, Ho M’s group has identified a critical groove formed by F41 and its surrounding amino acids located in a putative frizzled (FZD)-like cysteine-rich domain of GPC3 N-subunit and demonstrated that this groove is responsible for binding Wnt3a and activating Wnt signaling. Consistently, mutations in F41 or antibodies targeting the FZD-like domain can effectively suppress Wnt signaling and HCC growth [113], whereas antibodies against the C-terminal epitopes show no such effect [93]. These studies collectively imply that in certain HCC cells, it is the N-subunit of the GPC3 core protein, rather than the C-subunit or the HS chains in the C subunit, that is responsible for Wnt binding and pathway activation, thereby promoting HCC growth.

However, there is also some evidence showing that HS chains are essential for GPC3 to activate Wnt signaling. For instance, although Filmus J’s team has indicated that the HS chains of GPC3 are not required for Wnt binding; they demonstrate that the HS chains are responsible for direct interaction with FZD, the Wnt receptor, thereby facilitating the formation of the GPC3–Wnt–FZD complex and then triggering its endocytosis and subsequent signaling activation [114]. Gao W et al. have reported that the antibodies specifically targeting the HS chains of GPC3 interfere with the interaction between Wnt3a and GPC3, which in turn blocks Wnt3a/β-catenin signaling [115] and thus can inhibit the proliferation of HCC cells in vitro and in vivo. Additionally, they discovered that the HS chains mediate GPC3’s interaction with HGF and its promotive effects on HCC cell migration and motility. Antibodies against the HS chains can down-regulate HGF signaling, thus inhibiting HCC metastasis, an effect not observed with antibodies against the GPC3 protein [98]. Additionally, HS chains may be required for GPC3 to bind and activate FGF2 in HCC [97,116]. Therefore, current studies present conflicting results regarding the contribution of HS chains in GPC3-mediated pro-oncogenic effects, which may be due to distinct experimental conditions.

The impact of the convertase cleavage of its precursor on GPC3’s oncogenic effect has also been studied. Filmus J’s team has demonstrated that the GPC3 RR-AA mutant, which is resistant to convertase processing, retains the ability to interact with Wnt and activate its signaling cascade, thereby promoting the proliferation of HCC cells both in vivo and in vitro [12]. This suggests that convertase processing is not a mandatory step for GPC3 to activate Wnt signaling and stimulate HCC growth. However, Cheng W et al. show that the N-terminal proline-rich structural domain (p25-30) of GPC3 accounts for its interaction with IGF-II and IGF-1R, leading to the activation of the IGF-II/Erk pathway in HCC cells. The RR-AA mutation results in a significantly weaker interaction between GPC3 and IGF-II than the wild-type [85], indicating that convertase cleavage is necessary for GPC3 to effectively interact with IGF-II and initiate downstream signaling. Therefore, the necessity of convertase cleavage for GPC3’s pro-oncogenic activity appears to be selective, depending on the specific growth factors involved.

Then does the oncogenic effect of GPC3 require its attachment to the cell membrane? Evidence from Filmus J’s team indicates that while wild-type GPC3 promotes HCC cell proliferation, the GPC3ΔGPI mutant, which generates soluble GPC3, instead inhibits the proliferation of HCC cells (for more details, see Section 5.3) [82,117]. This finding suggests that the membrane-bound state of GPC3 is crucial for its oncogenic activity.

Thus, there are some studies presenting conflicting conclusions, especially regarding the role of the HS chains of GPC3. This variability may arise from the inherently heterogeneous nature of cancer cells. Even cancer cells from the same tissue type or different subpopulations within the single tumor mass can have different genetic backgrounds and oncogenic mechanisms. For example, the expression levels of GPC3 varies greatly among HCC cell lines from diverse origins, and the activation status of other oncogenic-related signaling pathways and the expression levels of related signaling molecules can also fluctuate significantly across different cancer cells. These variable factors contribute to the intricate complexity of the mechanisms by which GPC3 exerts its oncogenic effects.

### 5.3. GPC3’s Structural Features and Its Anti-Oncogenic Function

Based on the existing studies, convertase cleavage and the membrane-bound or soluble state seem to have a greater impact on the anti-oncogenic activity of GPC3, while the HS chains play a less critical role. As early as in 1998, Filmus J’s team found that membrane attachment but not HS chains is required for GPC3 to induce apoptosis in mesothelioma and breast cancer cells [11]. Later, Cat BD et al. further confirmed that the HS chains are not necessary for GPC3 to induce apoptosis in the above two types of cells. However, convertase processing is indispensable, in agreement with the observation that convertase processing, rather than the HS chains, is essential for GPC3 to interact with Wnt5a and activate its downstream non-canonical pathway, leading to apoptosis in both cell types [13].

Though GPC3 undoubtedly acts as an oncogene in HCC, Filmus J’s team has elucidated that soluble GPC3, achieved by expressing the GPC3ΔGPI mutant, significantly inhibited the proliferation of various HCC cell lines by the extensive blockade of multiple signaling pathways, including the canonical Wnt pathway and some growth factors [117]. Similarly, the direct addition of recombinant GPC3 protein to the cell culture medium of HCC cells does not promote cell proliferation but instead induces apoptosis in CD26^+^ HCC cells [118]. This study also demonstrates that the interaction between GPC3 and CD26 is independent of HS chains, further providing direct evidence that the soluble form of GPC3 can exert an inhibitory effect on at least a subset of cancer cells.

More evidence showing that soluble GPC3 may serve an anti-oncogenic purpose comes from the study in CCC. As mentioned earlier, despite being highly expressed in CCC, GPC3 functions to inhibit oncogenic processes. This is attributed to the fact that GPC3 in CCC is secreted in a soluble form, which impedes CCC cell growth by suppressing the IGF-II-signaling pathway [63]. These findings collectively highlight that the soluble form of GPC3 potentially exerts anti-oncogenic effects in certain cellular contexts.

**Table 2:** Relationship between GPC3’s structural features and its functions.

		Feature	HS	Convertase	Anchored	Soluble	N-Glycan
	Effectors						
Physiological function	(anti-) Hh		No [46], Yes [111]	Yes [112]	Yes [46]	No [46]	-
	(pro-) Unc5D		-	No [50]	-	-	Yes [50]
pro-oncogenic function	(pro-) canonical Wnt		No [82], Yes [114]	No [12]	Yes [117]	No [117]	-
	(pro-) IGF-II		-	Yes [85]	-	-	-
	(pro-) FGF2		Yes [97,116]	-	-	-	-
	(pro-) HGF		Yes [98]	-	-	-	-
anti-oncogenic Functions	(pro-) non-canonical Wnt		No [11]	Yes [13]	Yes [11]	-	-
	(anti-) IGF-II		-	-	-	Yes [63]	-
	(anti-) CD26^+^		No [118]	-	-	Yes [118]	-
	(anti-) canonical Wnt		-	-	-	Yes [117]	-

HS, GPC3 modified with HS chains; convertase, GPC3 cleaved by convertase; anchored, GPC3 anchored to the cell membrane; soluble, soluble GPC3; No, not required; Yes, required; -, not available.

## 6. Conclusions and Perspective

Above all, it is evident that GPC3 usually inhibits cell proliferation and/or promotes cell differentiation by negatively modulating various growth-factor-signaling pathways under physiological conditions. Interestingly, GPC3 plays a dual role in cancer, capable of either promoting or inhibiting tumor development. Despite the high frequency of GPC3 mutations in SGBS, data from cBioPortal (http://cbioportal.org (accessed on 2 April 2025)) indicate that the mutation frequency of GPC3 is quite low across different types of cancer. Thus, the disparity in GPC3’s role in distinct cancers is not primarily mutation-driven but rather seems to be associated with its structural features, as evidenced by the findings summarized herein. For instance, current evidence suggests that soluble GPC3 likely exerts an inhibitory effect in cancers, whereas membrane-bound GPC3 may trigger oncogenic effects by potentiating the binding of growth factors to their corresponding membrane receptors. In addition, due to the highly heterogeneous nature of cancer cells, GPC3 can exert pro- or anti-oncogenic effects via distinct mechanisms in different tumor cells, which, together with the complex tumor microenvironment, renders the biological effects of GPC3 in tumors intricate and unpredictable.

Therefore, further in-depth and meticulous studies are needed in the future to comprehensively reveal and clarify the biological functions of GPC3 and the underlying mechanisms in cancer occurrence and progression. Such knowledge is essential for the successful deployment of GPC3-targeted therapies in cancer treatment.

## Figures and Tables

**Table 1:** GPC3 protein levels in tumor tissues versus normal tissues.

Type of Cancer	Tumor vs. Normal	Reference
Rhabdomyosarcoma	high	[52,53]
Choriocarcinoma	high	[54]
Hepatocellular carcinoma	high	[55,56,57]
Lung squamous carcinoma	high	[58,59,60]
Yolk sac tumors	high	[61,62]
Ovarian clear cell carcinoma	high	[63,64]
Melanoma	high	[65]
Malignant mesothelioma	high	[66]
Gastro-esophageal adenocarcinoma	high	[67]
Pediatric solid embryonal tumors	high	[68]
Pancreatic ductal adenocarcinoma	high	[69]
Thyroid cancer	high	[70]
Lung adenocarcinoma	low	[71]
Breast cancer	low	[72]

## Data Availability

Not applicable.

## Abbreviations

The following abbreviations are used in this manuscript:

ACC	Adrenocortical cancer
AFP	Alpha-fetoprotein
BLCA	Bladder cancer
BM	Bone marrow
BMP	Bone morphogenetic protein
BRCA	Breast cancer
BWS	Beckwith–Wiedemann syndrome
CAF	Cancer-associated fibroblast
CCC	Clear cell carcinoma
CESC	Cervical cancer
CHOL	Bile duct cancer
COAD	Colon cancer
DLBC	Large B-cell lymphoma
EMT	Epithelial–mesenchymal transition
ER	Endoplasmic reticulum
ESCA	Esophageal cancer
ESCC	Esophageal squamous cell carcinoma
FZD	Frizzled
GBM	Glioblastoma
GC	Gastric cancer
GPC	Glypican
GPC3	Glypican3
GPI	Glycosylphosphatidylinositol
GLUT4	Glucose transporter4
HCC	Hepatocellular carcinoma
hESC	Human embryonic stem cell
HGF	Hepatocyte growth factor
Hh	Hedgehog
Hhex	Hematopoietically expressed homeobox
HNSC	Head and neck cancer
HS	Heparan sulfate
HSPC	Hematopoietic stem/progenitor cells
HSPG	Heparan sulfate proteoglycan
IGF-II	Insulin-like growth factor II
IGF1R	IGF receptor-1
IRS-1	Insulin receptor substrate-1
KGF	Keratinocyte growth factor
KICH	Kidney chromophobe
KIRC	Kidney clear cell carcinoma
KIRP	Kidney papillary cell carcinoma
LAML	Acute myeloid leukemia
LGG	Lower-grade glioma
LIHC	Liver hepatocellular cancer
LRP1	Low-density lipoprotein receptor-related protein-1
LUAD	Lung adenocarcinoma
LUNG	Lung cancer
LUSC	Lung squamous cell carcinoma
MAPK	Mitogen-activated protein kinase
MEF	Mouse embryonic fibroblast
MMP	Matrix metalloproteinase
OCCA	Ovarian clear cell carcinoma
OV	Ovarian cancer
PAAD	Pancreatic cancer
PCPG	Pheochromocytoma and paraganglioma
READ	Rectal cancer
PRAD	Prostate cancer
Ptc	Patched
SARC	Sarcoma
SGBS	Simpson–Golabi–Behmel syndrome
Shh	Sonic Hedgehog
SKCM	Skin cutaneous melanoma
STAD	Stomach cancer
SULF2	Sulfate esterase 2
TGCT	Testicular cancer
TGF-β	Transforming growth factor-β
THCA	Thyroid cancer
THYM	Thymoma
TIDE	Tumor Immune Dysfunction and Exclusion
TIMP-1	Tissue inhibitors of metalloproteinase 1
UCEC	Endometrioid cancer
UCS	Uterine carcinosarcoma
Unc5D	Uncoordinated-5 receptor D
V2	Variant 2
YAP	Yes-associated protein
